# Lower Urinary Tract Symptoms as an Indicator of Occult Neurologic Disease: A System-first Framework for Urologic Practice

**DOI:** 10.1007/s11934-026-01343-2

**Published:** 2026-06-02

**Authors:** Elizabeth Minnerath, Olga Khazen, Jalesh N. Panicker, Marcus J. Drake, Elise J. B. De

**Affiliations:** 1 Albany Medical College, Albany, NY USA; 2 Department of Uro‑Neurology, The National Hospital for Neurology and Neurosurgery, University College London NHS Foundation Trust, London, UK; 3 Department of Translational Neuroscience and Stroke, UCL Queen Square Institute of Neurology, Faculty of Brain Sciences, University College London, London, UK; 4 Department of Neurological Urology Surgery, Imperial College London, London, UK

**Keywords:** Occult neurologic disease, Lower urinary tract symptoms, Urodynamics, Multidisciplinary care

## Abstract

**Purpose of Review:**

Lower urinary tract symptoms (LUTS) may precede or accompany the other symptoms of neurologic disease and may be the presenting clinical manifestations of central, peripheral, or autonomic nervous system dysfunction. In clinical practice, urologists are often the first specialists to evaluate these patients, potentially before underlying neurologic disease has been identified. Early recognition of neurologic contributors to LUTS may help prevent diagnostic delay. This review aims to provide a symptom-based framework that highlights common urologic presentations associated with underlying neurologic disease, along with urodynamic findings of concern, to support earlier recognition of neurological disease in patients presenting with LUTS.

**Recent Findings:**

Recent work has characterized neurologic diseases associated with LUT dysfunction using disease-based frameworks. Despite this, gaps persist in routine urologic evaluation, including inconsistent screening for neurological symptoms and limited incorporation of focused neurologic history and examination. Certain urodynamic patterns are increasingly recognized as suggestive of neurological pathology but are not consistently interpreted within this context. Barriers to timely neurologic evaluation, including limited specialist availability and prolonged wait times, further contribute to diagnostic delay. As a result, there is growing recognition of the need for urologists to identify clinical features within LUTS presentation that should prompt consideration of neurologic disease, though practical strategies for integrating these insights into routine care remain limited.

**Summary:**

A symptom-based framework for evaluating LUTS can enhance early detection of neurologic disease in urologic practice. Urologists and pelvic health specialists should have a broad understanding of what clinical characteristics associated with LUTS should trigger concern for underlying neurological disease and should always consider neurological disease in the differential diagnosis. This review provides a practical, clinically oriented overview of neurologic disease in LUTS assessment, including guidance on screening questions, initial testing considerations, urodynamic interpretation, and referral, particularly in the settings with limited neurology access. Algorithms are provided to facilitate early recognition of occult neurological disease within routine urologic practice.

**Supplementary Information:**

The online version contains supplementary material available at 10.1007/s11934-026-01343-2.

## Introduction

Lower urinary tract symptoms (LUTS) are among the most common reasons for urologic consultation [1] and are traditionally approached by urologists through an anatomic or organ-specific lens. However, micturition is a complex neurophysiologic process requiring coordinated central, peripheral, and autonomic (sympathetic and parasympathetic) control [2]. As opposed to other autonomic functions in the body such as the cardiovascular system, urinary control is bimodal (storage and emptying) and depends on learned behavior. The pontine micturition center (PMC) in the pons can be thought of as the activation switch and the cortical level periaqueductal gray (PAG) as the flipper of the switch. The PAG receives input from the anterior cingulate gyrus (conscious attention to sensation) and the prefrontal cortex (the center of cognitive and socially appropriate behavior) [3, 4].

Ultimately, the coordinated signals (described in detail in "Neurological Control of the Lower Urinary Tract" Section) produce a detrusor contraction with synergistic relaxation of the urethral sphincters, enabling effective micturition. Disruption at any level of the nervous system may manifest initially and sometimes predominantly as LUTS.

LUTS may precede more overt neurologic deficits by months or years, may be the most bothersome symptoms driving evaluation, or may present as one component of a symptom array across a wide range of neurologic conditions. For many patients, the urology visit represents their first specialty evaluation. Urologists who recognize patterns suggestive of underlying neurologic dysfunction are in a unique position to initiate focused screening and diagnostic evaluation and assess the priority and appropriateness of neurology referral. Diagnosis allows: prevention of unnecessary urologic surgeries (sling, prostate surgery), clarification of prognosis and suitability for clinical trials (multiple system atrophy), and initiation of disease modifying medical (e.g. multiple sclerosis), preventative (small vessel disease), or surgical intervention (e.g. shunt in normal pressure hydrocephalus) [5, 6]. In practice, timely neurologic consultation is often limited by access constraints, further amplifying the importance of the urologists’ role as part of the multidisciplinary team. The aim of this manuscript is to provide practical, efficient tools that can be integrated into routine urologic workflows, improving outcomes beyond LUTS by treating the underlying cause [5–8].

**Fig. 1:**
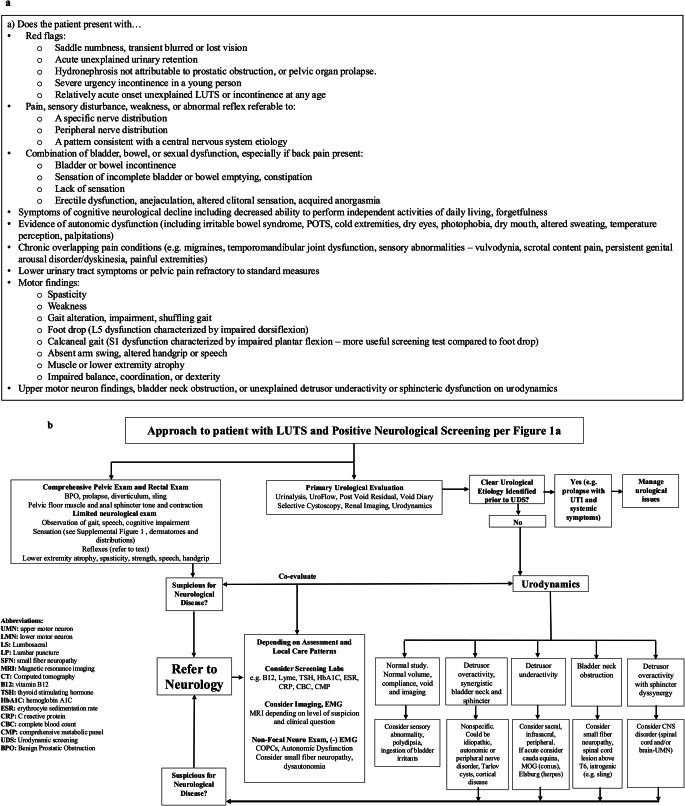
Identification of occult neurological disease (OND) in patients presenting with lower urinary tract symptoms. (**a**) Screening symptoms and signs that prompt concern for OND, consideration for referral to neurology (**b**) algorithm for appropriate multidisciplinary workup

Our prior reviews characterize the frequency and presentation of LUT dysfunction across central, peripheral, and autonomic neurologic conditions; here the reader can find more of a neurological disease framework [3, 5, 7] or a focused urologic tutorial [6]. The *current* review is comprehensive and written for urologists and pelvic health specialists – explaining neurophysiology and diagnoses relevant to LUTS but highlighting presenting symptoms, signs and urodynamics as practical tools for the non-neurologist to identify occult neurological disease (OND) in patients with LUTS. Clinically accessible screening tools, key indicators in urodynamic testing, and a broad multidisciplinary algorithm are presented. Figure 1a provides screening questions and exam findings suspicious for occult neurologic involvement and Fig. 1b is an algorithm for appropriate urologic workup. Table 1 as well as Supplemental Tables 1 and 2 provide guidance on neurologic levels associated with specific LUTS and urodynamic examples of concerning urodynamic patterns.

## Neurological Control of the Lower Urinary Tract

During storage, the bladder fills while the detrusor muscle remains relaxed. The sacral parasympathetic neurons (S2-S4) are suppressed through spinal interneurons. These interneurons integrate mild stretch afferent signals to the sacral spinal cord (S2-S4) and PAG, integrating bladder fullness with input from the prefrontal cortex (what is socially appropriate) to maintain continence. Sympathetic fibers from T11–L2 travel via the hypogastric nerve (of note this nerve is visible medial to the ureter during pelvic surgery and should be protected) and then intermingle with the parasympathetic fibers in the inferior hypogastric plexus (also vulnerable during pelvic surgery). The sympathetics relax the detrusor (β3 receptors) and contract the internal urethral sphincter (α1 receptors). At the same time, somatic fibers from ventral roots of S2–S4 originating in Onuf’s nucleus travel through the pudendal nerve to keep the external urethral sphincter tonically contracted.

As the bladder fills, the efferent signal becomes stronger. Micturition can be delayed by higher level integration above the PMC (cortical lesions such as stroke or Lewy body dementia can alter this integration). Once the PMC is released from the tonic inhibition of higher brain centers (the switch is flipped), voiding occurs. The PMC coordinates bladder emptying via three projections that descend in bulbospinal reticulospinal pathways to the spinal cord. These pathways can be interrupted by disease impacting the spinal cord: (1) the PMC inhibits sympathetic neurons in the intermediolateral column (T11–L2) of the thoracolumbar cord, relaxing the bladder neck/internal sphincter directly and also inhibiting the spinal sympathetic interneurons that have been supporting tonic activity in Onuf’s nucleus; (2) it directly inhibits the somatic motor neurons in Onuf’s nucleus, allowing the EUS to relax via the pudendal nerves; (3) a moment later the bladder will contract as the PMC excites sacral parasympathetic neurons (S2–S4) to the detrusor. Parasympathetic fibers from the ventral roots of S2-S4 travel via the pelvic splanchnic nerves (a.k.a. “pelvic nerve”, “nervi erigentes”) through the inferior hypogastric plexus then the vesical plexus, entering close to the ureters, and release acetylcholine onto M3 receptors, causing detrusor contraction. These peripheral pathways are vulnerable during pelvic surgery (e.g. abdominoperineal resection or uterosacral ligament vault suspension) and also in autonomic or peripheral neuropathies. A note on the bladder neck: bladder neck relaxation during voiding is mediated primarily by withdrawal of α-adrenergic sympathetic tone under pontine control, with parasympathetic signaling facilitating smooth muscle relaxation via local inhibitory neurotransmitters rather than direct active dilation.

## Neurologic Diseases which may Present with LUTS

The following sections discuss common neurologic diseases which may present first to a urology clinic with LUTS, potentially before the neurologic condition is diagnosed, or even suspected. The Urologist should be particularly aware of multiple sclerosis (MS) and related neuro-inflammatory disorders, multiple system atrophy (MSA), Parkinson’s disease (PD), normal pressure hydrocephalus (NPH), dementia, structural spinal cord lesions [6], and small fiber neuropathy (SFN) [9] as the potential for LUTS to precede or call attention to other neurological symptoms is significant.

### Central Neurologic Disorders

#### Multiple Sclerosis and Related Neuroinflammatory Disorders

Multiple Sclerosis (MS), Neuromyelitis Optica Spectrum Disorder (NMOSD), and Myelin Oligodendrocyte Glycoprotein Antibody-Associated Disease (MOGAD) are neuroinflammatory disorders characterized by immune-mediated axonal damage throughout the CNS. All can cause transverse myelitis with spinal cord inflammation and axonal injury. The average age of onset is similar for these disorders, with mean ages of 20–30, 40.3, and 30 years respectively, and typical time from symptom onset to diagnosis is 1–2 years [10–15]. Patients can experience both storage and voiding symptoms depending on the location of the lesion.

MS primarily affects the brain (demyelinating plaques) and optic nerves, but transverse myelitis of the spinal cord occurs in 25%. Nearly 90% of patients with MS experience LUTS; they typically worsen with disease duration and spinal cord involvement and have been reported to appear up to 8 years after diagnosis [7, 16]. Patients most commonly experience storage symptoms such as urinary urgency (62–65%), frequency (50%), urge incontinence (45%), and nocturia (35%) [17, 18], but voiding symptoms (incomplete emptying, straining, weak stream) can be present secondary to detrusor external sphincter dyssynergia (DESD) or detrusor underactivity (DO) depending on the level of spinal cord involvement. Incomplete emptying can lead to recurrent urinary tract infection (UTI) [16, 19, 20]. Both voiding and storage symptoms are reported in up to 50% [7]. Erectile dysfunction (53%) and bowel dysfunction (40%) can be present early in disease [21]. Due to the inflammatory nature, history may include an abrupt onset of symptoms with subsequent improvement. Neurological symptoms may include monocular vision loss, blurred or double vision, sensory loss, weakness, ataxia, sensory loss of the face, arm, legs, pins and needles, gait disturbance, and weakness.

NMOSD is a debilitating group of syndromes associated with aquaporin-4 immunoglobulin G antibodies (AQP4-IgG) affecting the optic nerve, brainstem, and spinal cord. Patients may present with optic neuritis, myelitis, unexplained and intractable hiccups, and nausea [22]. NMOSD causes transverse myelitis more often than MS and involves more longitudinal extension within the spinal cord, leading to storage and voiding symptoms in 78–83% of patients [7]. Urinary retention is the most common symptom in this group [23]. Urodynamics demonstrates DO and DESD in over one third of cases [24].

MOGAD affects myelin oligodendrocyte glycoproteins (MOG) on myelin sheaths. Patients may present similarly to MS and NMOSD with optic neuritis and transverse myelitis but may also have acute disseminated encephalomyelitis (ADEM) and inflammatory demyelinating lesions anywhere in the CNS [25]. Diagnosis of MOGAD is by MOG antibody, which can be negative early in disease, as can MRI. Patients may experience urinary retention as an early sign of MOGAD [26] as the conus is particularly susceptible to injury in this condition. Overall LUT dysfunction is present in 28–59% of patients and whereas often the motor dysfunction improves, the urinary symptoms can remain [27].

Urodynamic findings in neuroinflammatory disease depend on the level affected (Table 1, Supplemental Table 1a-d). Higher level lesions of the neuroaxis would result in detrusor overactivity (DO) with or without DESD (Table 1, Supplemental Table 1a-b), decreased capacity, and over time possible decreased compliance (due to chronic mechanical stress and intermittent ischemia, which activate cellular repair and fibrosis pathways). Any level of the CNS is possible in all three, for example MOGAD is known for impacting the conus, which would lead to atonic bladder. In MS, as disease progresses, it is well known that urodynamic tracings change. Prompt urodynamic testing is recommended in the case of change in symptoms, and interval studies should be undertaken in concerning patterns such as poor compliance or vesicoureteral reflux [28].

**Table 1:**
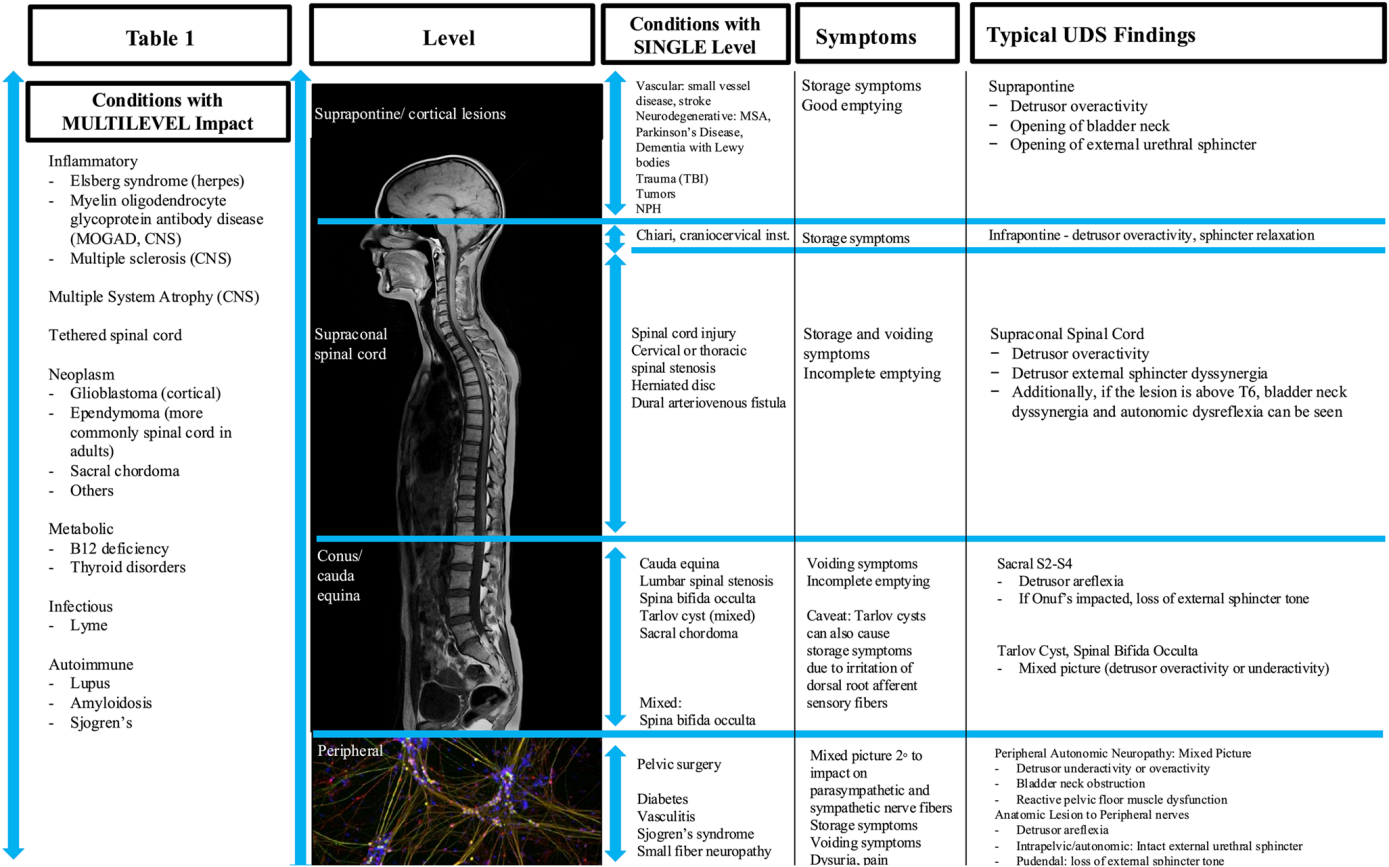
Categorization of neurological conditions by level of impact on central and peripheral nervous system. Lower urinary tract symptoms and expected urodynamic findings by level. Peripheral nerve image: courtesy https://nigms.nih.gov/image-gallery/3263 and Stephen Dalton, University of Georgia

#### Parkinsonian Disorders

Whereas LUTS are highly prevalent in PD, affecting between 27 and 85% of patients [7, 29–31], it is rare to see Parkinson’s as OND presenting with LUTS. Motor symptoms are typically present at diagnosis (tremor, rigidity, bradykinesia, postural instability) occurring at a mean age of 55–65 years old [32]. Parkinson’s disease is linked to MSA, Dementia with Lewy Bodies (DLB) and pure autonomic failure (PAF) in that they are all synucleinopathies – misfolded alpha-synuclein is abnormally deposited in cellular locations and patterns that lead to the distinct disorders. In Parkinson’s disease, degeneration of subcortical and cortical regions leads to disruption of dopamine D1-mediated GABAergic direct pathways, which is thought to contribute to impaired bladder control, reduced inhibition of the micturition reflex, and overactive bladder symptoms [7, 30]. The most common symptom PD patients experience is nocturia, followed by urinary urgency and increased frequency. Though symptoms may be present early in the disease course, they are exacerbated by disease progression. UTI is a debilitating and common complicating of LUT dysfunction in PD patients, most often in the setting of incomplete emptying [33].

The most common urodynamic findings early in PD include DO and low PVR due to loss of inhibition of the basal ganglia on the PAG. In late PD, bladder underactivity and high PVR occur mainly secondary to PMC dysfunction, as well as impaired autonomic (parasympathetic) input. Dysfunction of the external sphincter (pseudodyssynergia, Supplemental Table 2a) is now thought to play a more minor role but can show as a dilated prostatic urethra during voiding [7, 34–37]. Normal to poor bladder compliance (due to repeated involuntary contractions over time with cyclic ischemia and fibrosis) can be seen.

In contrast to PD, MSA is more likely to present with LUTS, with symptom onset at mean ages of 55–60 years old and time to diagnosis typically around 3 years [38, 39]. If affects 8 per 100,000 over age 40 and impacts genders equally. MSA is a devastating and progressive neurodegenerative disorder in which α-synuclein deposits impact the oligodendroglial cells, disrupting myelination and neuronal support. This affects central autonomic centers (brainstem, PMC, and PAG), spinal autonomic neurons, and sometimes peripheral autonomic fibers. Patients present with parkinsonism, cerebellar ataxia, slurred speech, sleep problems, upper motor neuron (UMN) pyramidal signs [40, 41], hypotension, cardiovascular dysfunction [42]. LUTS (urinary urgency with or without incontinence, nocturia, hesitancy, urinary retention) are common at presentation, are observed in 90% of cases overall [41, 43]. LUTS have been reported to precede motor symptoms by an average of 2.8 years in 20%In 20%, LUTS have been reported to precede motor symptoms by an average of 2.8 years [40, 41, 44]. For many men, erectile dysfunction is an early feature. Urinary retention can be a presenting feature [40]. In retrospect, 40% of men with MSA who presented with LUTS were misdiagnosed with BPH and had a poor outcome after prostate surgery [45].

Urodynamic findings of an open bladder neck during filling (Supplemental Table 2d) is present in 53% of patients with MSA and in none with PD [46] and is the most suspicious urodynamic finding. An open prostatic urethra may be seen during voiding. Detrusor overactivity (33–100%) and uninhibited external sphincter relaxation (33%) are similar to PD [46–48]. DESD is present in 47%. Detrusor underactivity is seen in in 71% of women and 63% of men with MSA (underactivity can be present in Parkinson’s but is less common [46]). Elevated post void residuals are present more commonly early in MSA versus PD and worsen over time. Impaired bladder sensation and poor compliance can also occur [34, 48], as variable findings can be seen depending on the stage of disease. Detrusor underactivity and external sphincter electromyography (EMG) abnormalities (degeneration of Onuf’s nucleus) suggest later stage and/or spinal involvement [47].

#### Dementias – NPH and other Dementias

The most common reported symptom in dementia is incontinence due to DO, which occurs secondary to decreased inhibition of the PMC (and therefore of the bulbospinal reticulospinal micturition reflex) [49]. LUTS are most common in patients with Lewy Body Dementias (DLB) and vascular dementias (80–90%) compared to frontotemporal and Alzheimer’s dementia (25–40%) [49] due to impact on regulation of the PMC.

In DLB, α-synuclein deposition is primarily neuronal (Lewy bodies and neurites) and affects cortex, limbic structures, basal ganglia, and brainstem/autonomic centers. Symptoms include early dementia, parkinsonism, autonomic dysfunction, and LUTS with mean age of onset at 68 years old and mean age of diagnosis at 72 years [50]. It presents similarly to PD, with additional psychiatric symptoms and memory impairment preceding the motor symptoms. LUTS typically present later in the disease with the most reported symptoms being nocturia and urgency urinary incontinence [7, 51, 52]. Urodynamics show DO with minimal PVR [51]. Additional findings in dementia patients can include decreased compliance and reported instances of decreased sphincter relaxation, likely due to impaired coordination of the PMC and basal ganglia control [37].

Vascular dementia is a broad term involving a broad spectrum of vascular cognitive impairment, including stroke, commonly occurring in individuals above 65 years old [53]. However, it most often is used to refer to small vessel ischemic damage affecting subcortical white matter. This is a more subtle form of vascular disease that can lead to frontal subcortical disinhibition of the PMC, leading to urgency, increased frequency, and urgency incontinence before significant cognitive decline, with worsening symptoms as white matter changes increase [54, 55]. Urodynamic studies are mixed, showing DO [55] or even underactivity with decreased sphincter contractability and relaxation correlating with the severity of ischemia [37, 56].

Normal pressure hydrocephalus (NPH) is a mechanistically distinct form of dementia presenting with a triad of gait disturbances, cognitive impairment, and urgency urinary incontinence, primarily occurring in individuals after age 60 [57]. Enlargement of the brain ventricles occurs despite a relatively normal cerebrospinal fluid pressure, compressing nearby frontal and periventricular white matter tracts, decreasing cortical inhibition to the PAG and PMC. One study found that the most common and bothersome LUTS in patients with new-onset NPH were nocturia and urgency incontinence [58]. Urinary urgency has been demonstrated to be an earlier manifestation with incontinence developing as the disease progresses [58, 59]. UDS findings show predominantly DO, with some instances of bladder outlet obstruction, and detrusor underactivity in a very small percentage of patients [58, 60].

#### Cerebrovascular Disease

While vascular dementia and cerebral small vessel disease produce lower urinary tract symptoms through chronic suprapontine disinhibition, cerebrovascular disease from focal infarction represents a distinct mechanism in which LUT dysfunction varies according to lesion acuity, laterality, and involvement of descending motor pathways - severity and location of the UMN damage [37]. Risk of cerebrovascular disease begins in middle age, increasing to peak risk around 70 years of age [61].

#### Spinal Canal Stenosis

Whereas acute spinal cord injury is unlikely to present as OND with LUTS, “spinal stenosis” involves chronic narrowing of the spinal canal compressing the cord or nerve roots secondary to degenerative changes or spondylolisthesis. Degenerative changes commonly present around the mean age of 64–65 years old [62]. Compression of the spinal cord may produce neurological deficits below the level of lesion, including gait disturbance, sensory changes, and LUTS. The most common sites of stenosis are the cervical and lumbar spine. Patients may present with extremity pain and/or paresthesia, and LUTS typical of the applicable spinal cord level (specifically underactivity, incomplete bladder emptying, overflow incontinence and frequent UTIs below S2, and hesitancy, urgency incontinence, nocturia at higher levels [63]). Back pain can be absent if only central compression occurs.

Cauda equina syndrome is most commonly recognized as an acute presentation characterized by saddle anesthesia, poor detrusor contraction, external urethral sphincter laxity, loss of anal tone, and stress or overflow incontinence due to compression of the cauda equina, the bundle of lumbar and sacral nerve roots (typically L2-S5), but can also be chronic in the setting of spinal stenosis or slow growing spinal tumors. Urodynamic testing for stenosis at this level shows increased bladder capacity and PVR, preserved or increased compliance, and low flow rate [63, 64], and higher levels would be expected to match the spinal cord level, as shown in Supplemental Table 1c. As spinal stenosis occurs typically in older individuals, anatomic factors such as prostatic obstruction or pelvic organ prolapse may play a role.

#### Craniocervical Junction Disorders (Chiari Malformation, Craniocervical Instability)

Disorders of the craniocervical junction, including Chiari malformation and craniocervical instability, impact the level between the pons and the spinal cord, best classified as *infrapontine* lesions, involving the lower brainstem (medulla), cervicomedullary junction, and upper cervical spinal cord. They may impair brainstem and upper cervical cord control of urination, leading to LUT and autonomic symptoms that can precede overt neurologic diagnosis. Cervicomedullary compression interferes with descending inhibitory control over the PMC leading to DO on urodynamic testing. The reticulospinal pathways from the PMC to Onuf’s nucleus are preserved, at least early, and in the absence of significant cervical myelopathy or syringomyelia, DESD is absent [3, 65]. Symptoms worsen particularly with Valsalva, exertion, sustained upright posture, or neck movement (e.g. looking up) and are accompanied by headache, neck pain, dysphagia, upper‑extremity paresthesias, or other autonomic features. Routine brain or spine MRI may be normal, necessitating targeted posterior fossa or dynamic craniocervical imaging when suspicion remains high.

### Sacral Lesions

#### Spinal Bifida

Spina bifida may cause disruption to the spinal cord and can be diagnosed prenatally, immediately upon delivery, or be missed, even into adulthood (spina bifida occulta) [66, 67]. It has a 12% prevalence radiographically but only 1/1000 of the population has neurological sequelae [68]. Patients can experience lower extremity paresthesia, weakness or spasticity, unexplained gait disturbance, lower extremity atrophy (may have different shoe sizes), back pain, hairy tuft, scoliosis, bowel dysfunction and LUTS [5]. It can be associated with tethered cord syndrome, in which case history may reveal onset of symptoms during teen years or other growth spurts. Urodynamic findings are varied, especially if tethered cord is identified, as the tension can lead to a mixed upper and lower motor neuron picture (normal, increased or reduced capacity, compliance, and detrusor activity with or without DESD depending on the extent of the tethered cord) [37]. Detrusor overactivity has been reported in 42% and poor compliance in 67% [69]. If only the sacral nerve roots are involved without tethering, detrusor underactivity with a compliant bladder would be expected.

#### Sacral Cysts

Tarlov perineural spinal cysts (TCs) are dilations that form within the sensory nerve root sleeves, where cerebral spinal fluid (CSF) extends distally and can accumulate. They are typically asymptomatic until the fourth to sixth decade of life [70]. They occur most often in the sacral spine where the nerve roots are under the highest hydrostatic pressure and lack enclosing vertebral foramina, and are more common in women and in those with connective tissue disorders. As CSF accumulates between the perineurium and endoneurium, it stretches traversing axons and displaces them laterally into cyst walls. Axons under pressure can fire spontaneously, triggering neuropathic symptoms, and ruptured axons cause distal Wallerian degeneration, denervating peripheral targets. They are present in 15% of individuals and thought to be symptomatic in 15% of cases, most commonly causing sacral dermatomal neuropathic pain (e.g. S2 pelvic dermatomal pain radiating to the inner calf), as well as bladder, bowel, and sexual dysfunction (e.g. persistent genital arousal disorder (PGAD)) [71]. Urinary symptoms are reported in more than 90% of patients, and sacral root injury has been objectively demonstrated in nearly 60% of patients through pelvic neurophysiology testing [72]. Urodynamic patterns can be mixed depending on location of the cyst along the nerve root. Urinary symptoms and objective sacral root injury is demonstrated in most patients with midline meningeal sacral cysts [73]. Sacral cysts are not consistently reported by radiologists [72] and therefore it is helpful to look oneself for cystic structures that are bright on magnetic resonance T2 imaging of the lumbosacral spine and pelvis.

#### Sacral Chordoma

Sacral Chordoma typically presents at a mean age of 62.7 years old [74] with local pain and progressive neurologic or pelvic symptoms due to a slow-growing tumor arising from notochord remnants in the sacrum. It can be worse with sitting, and radiate to the buttocks or posterior thighs. Involvement of the S2-S4 nerve roots can cause LUTS and radicular lower extremity pain or weakness. A palpable fullness may be appreciated on rectal exam. Due to its slow progression, time from symptom onset to diagnosis can be prolonged, with median time of 2 years [75]. Early diagnosis improves prognosis.

#### Infectious Sacral Radiculopathies (HSV, VZV)

HSV-2 can cause sacral radiculitis affecting the S2-S4 nerve roots (Elsberg syndrome), producing subacute or acute lower urinary tract symptoms [76]. Acute urinary retention or acute voiding dysfunction with pain can occur, as well as bowel or sexual dysfunction. Visible genital lesions are not always present. Herpes Zoster–related sacral radiculopathy can present prior to the rash and can be in association with dermatomal pain out of proportion to exam, typically presenting in older populations [77]. Sensation and detrusor contractility can be reduced in both entities. HSV and VSV should be suspected in acute urinary retention, severe LUT symptoms with pain, perineal sensory complaints, and dysuria with sterile urine, especially when associated with a rash.

### Peripheral Neurological Disorders – Peripheral Neuropathies

Peripheral neuropathies are a heterogeneous group of disorders affecting peripheral nerves. They vary in etiology, nerve types involved, and pathophysiology. Peripheral nerves are classified by diameter, myelination, and function. Large fibers (Aα, Aβ) are thickly myelinated, and function for proprioception, vibration, and motor control. Small fibers (Aδ, C) are thinly myelinated, and mediate pain, temperature, and autonomic function. The standard neurological reflex exam and EMG/nerve conduction study (NCS) can assess the myelinated large nerve fibers but not small fibers. Whereas a radiculopathy (nerve root compression) and focal neuropathy (e.g. carpal tunnel syndrome) are technically considered peripheral neuropathies, this section will focus on diffuse systemic neuropathies.

Etiologies of diffuse peripheral neuropathies include metabolic (e.g. Diabetes Mellitus), toxic (alcohol, chemotherapy), nutritional (e.g. vitamin B12 deficiency), autoimmune (e.g. Guillain–Barré Syndrome, Chronic Inflammatory Demyelinating Polyradiculoneuropathy (CIDP)), genetic (e.g. Charcot–Marie–Tooth Disease (CMT)), and infectious (e.g. Lyme disease). They have varying impacts on sensory, motor, autonomic and mixed peripheral nerve fiber types.

Peripheral neuropathies typically present with voiding dysfunction and worsen with disease progression. Patients with severe bladder neck dysfunction may experience retention [9, 78]. Diabetic neuropathy is a common peripheral neurological disorder, with less common considerations relating to autoimmune, inflammatory, or inherited peripheral neuropathies such as SFN, Guillain-Barre Syndrome, CMT, and CIDP. Generally, UDS can show anything from detrusor overactivity to increased capacity and compliance, impaired sensation, and decreased detrusor contractile activity, to abnormalities of the bladder neck, all due to varying autonomic sympathetic and parasympathetic nerve dysfunction [37].

#### Diabetic Neuropathy

‘Diabetic cystopathy’ is a complication of diabetic peripheral neuropathy, with onset correlating with duration of disease and glycemic control [79]. Glucose is converted to sorbitol via the aldose pathway, which accumulates within nerve cells, ultimately leading to oxidative injury. Diabetes is the most common cause of small fiber neuropathy (next section) in its early stages. The picture can become mixed large fiber as damage continues [80]. Detrusor underactivity is the classic concept of diabetic neuropathy seen in later stages of disease but any manifestation of peripheral nerve injury can be seen, for example bladder neck obstruction secondary to loss of inhibition of sympathetic nerves at the bladder neck [6, 9, 80], DO (55%) impaired detrusor contractility (23%), detrusor areflexia (10%), and bladder outlet obstruction (36%, all men) [80].

#### Small Fiber Neuropathy

While SFN is less commonly discussed, its recognition is an emerging diagnosis in urology, urogynecology, pain management, and neurology [81, 82]. It is a pathologic pattern of neuropathy defined by damage to small nerve fibers (Aδ, C fibers) presenting at a mean age of 54–55 years, with time from onset to diagnosis an average of 4.8 years [82, 83]. SFN represents a heterogeneous group of etiologies rather than a distinct condition. Since SFN involves abnormalities of the Aδ and C fibers which are responsible for pain, temperature, and autonomic signal transmission, patients present to the Urology clinic with voiding dysfunction and/or pelvic pain [81, 82], These fibers impact urinary symptoms (incomplete emptying, dysuria, increased frequency/urgency, hesitancy), urodynamics findings (detrusor under- or overactivity, bladder neck dysfunction), sexual dysfunction, PGAD, and chronic pelvic pain [9, 82, 84, 85]. Emerging evidence links SFN to autonomic bladder dysfunction in patients with complex chronic pelvic pain syndromes and accompanying systemic conditions [86–89]. SFN may be suspected based on the additional non-urologic complaints, for example anorectal pain, defecatory dysfunction, irritable bowel syndrome, impaired gastrointestinal motility, nausea, heartburn, migraines, tinnitus, temporomandibular joint dysfunction, fibromyalgia, chronic back pain, extremity pain, loss of hair on extremities, palpitations, anxiety, asthma, and orthostasis, which occur secondary to dysfunction of the small unmyelinated nerve fibers. Collectively, these symptom patterns are referred to as chronic overlapping pain syndromes and/or autonomic dysfunction.

Clinically, SFN will be distinguished from CNS or large nerve fiber causes of autonomic dysfunction by a non-focal neurologic examination and normal electromyography. Definitive diagnosis often involves skin biopsy demonstrating decreased intraepidermal nerve fiber density. Additional testing may include confocal microscopy, autonomic testing for dysautonomia, and laboratory evaluation for reversible etiologies [90–95]. Recognition of SFN allows for therapy targeted to neuropathic pain and other dysfunction, and in some cases, reversal of underlying etiology (e.g. Lyme disease, vitamin B12 deficiency, Celiac disease). Key urodynamic findings that lead to suspicion of SFN in the setting of the above review of systems include bladder neck obstruction or underactive bladder [9, 96, 97].

### Autonomic Neurological Disorders

Although many neurological diseases impact the *function* of the sympathetic and parasympathetic nerves to the bladder, autonomic neurological disorders involve *impairment* of the autonomic nerves themselves. These disorders therefore commonly present with symptoms in multiple systems (e.g. gastrointestinal, cardiovascular, ophthalmic, and urinary). Autonomic neurological disorders can arise from peripheral nerve damage (e.g. Diabetic Autonomic Neuropathy, pure autonomic failure), CNS dysfunction (e.g. MSA, PD), or both (e.g. Amyloidosis). Peripheral lesions affect postganglionic fibers (orthostatic hypotension, bladder, or GI dysfunction) and central lesions disrupt autonomic integration in the brain or spinal cord (affecting heart rate, blood pressure, and temperature regulation). There can be predominant involvement of the autonomic nervous system in both central (e.g. MSA) and peripheral (e.g. SFN and Diabetic neuropathy) neurologic disorders which impacts the bladder via sympathetic and parasympathetic dysfunction.

#### Neurodegenerative Disorders

Autonomic dysfunction is highly prevalent in neurodegenerative disorders including MSA, PD, DLB, discussed in "Parkinsonian Disorders" Section above, and pure autonomic failure (PAF). The underlying pathophysiology is thought to be the deposition and accumulation of alpha-synuclein throughout the central and peripheral nervous system, resulting in dysfunction of nerves affected [7]. Patients often present with cardiovascular symptoms, such as orthostatic hypotension, which may distract from the urinary symptoms. Patients presenting with PAF (orthostatic hypotension, bladder and bowel dysfunction, and impaired sweating) often report LUTS (96%), overactive bladder symptoms (92%), voiding symptoms (76%), PVR > 100 (31%), and nocturnal polyuria (86%) [98]. They are at increased risk (33%) for phenoconversion to other neurodegenerative diseases (MSA, PD, or DLB) [99, 100], with urinary dysfunction serving as an important predictor [7, 101]. Early recognition of patients presenting with multisystem autonomic dysfunction along with LUTS is critical.

#### Postural Orthostatic Tachycardia Syndrome

Postural Orthostatic Tachycardia Syndrome (POTS) presents as excessive increases in heart rate without significant drop in blood pressure during positional changes from laying to standing. Patients may experience dizziness, fainting, chest pain, palpitations and several other non-cardiovascular symptoms leading to orthostatic intolerance [96].The mechanisms are thought to be peripheral autonomic denervation and venous pooling, excessive norepinephrine, hypovolemia, or a broader small fiber neuropathy. Presentation occurs at a mean age of 14 years old, is more common in young females, and significant delays in diagnosis lead to a mean age at diagnosis of 30 years [102]. Patients may report LUTS such as nocturia, increased urinary frequency, and urgency due to associated systemic autonomic dysfunction [7, 96, 103]. Urodynamic findings are DO in 50% and detrusor underactivity in 50% [96]. Other reviews suggest UDS findings consistent with decreased bladder sensation and contractility, and functional obstruction [7].

#### Rare Mixed Autonomic Disorders

There are some rare mixed disorders that can impact autonomics such as Lambert-Eaton Syndrome (LES), which is a neuromuscular junction disorder affecting pre-synaptic calcium channels. It impacts the neuromuscular junction as well as postganglionic autonomic nerve endings. It has a bimodal age of onset, with peaks around 30 and 60 years, and diagnosis is often delayed by 6 to 12 months [104, 105]. Patients present with a constellation of autonomic symptoms, such as dry mouth, proximal muscle weakness, and increased urinary frequency. Urinary symptoms have been shown to precede other characteristic symptoms by years [7], however, they are rare and not well reported in the literature [106].

There are some rare mixed disorders that can impact both central and peripheral autonomic pathways by mechanisms such as central degeneration, postganglionic fiber loss, SFN, autoantibody-mediated synaptic blockade, and toxic deposition. Examples include Amyloidosis (toxic amyloid deposition damaging central nuclei and peripheral ganglia), Familial Dysautonomia (genetic defect affecting brainstem and peripheral neurons), MSA**/** PD with autonomic failure ( "Parkinsonian Disorders" Section, degeneration of central autonomic nuclei with secondary peripheral denervation), and Autoimmune Autonomic Ganglionopathy (AAG).

AAG is a *treatable* cause of acute to subacute urinary retention in association with other autonomic symptoms (orthostatic hypotension, dry mouth and dry eyes, photophobia, constipation or GI dysmotility, abnormal sweating), typically following an immune challenge. The mechanism involves autoantibodies against nicotinic ganglionic receptors causing widespread autonomic failure, and diagnosis is by positive serology for ganglionic acetylcholine receptor antibody (gAChR-Ab) and the patients’ response to intravenous immunoglobulin therapy [107].

## Muscular Disorders

Rarely LUTS can precede systemic symptoms in primary muscular diseases such as myotonic dystrophy, dystrophinopathies (e.g. Duchenne/Becker), and smooth muscle myopathies. Onset can range from infancy to late adulthood, with diagnosis up to 2 years after onset [108, 109]. These urinary symptoms arise from early involvement of the bladder’s smooth muscle or sphincter dysfunction before overt skeletal muscle weakness is clinically evident.

## Symptom-first Entry Point: A Practical Algorithm

In routine clinical practice, patients present with symptoms rather than formal diagnoses, making a symptom-first framework practical in urologic settings. Recognizing patterns suggestive of neurologically-mediated LUTS requires attention to symptom clusters that extend beyond lower urinary tract complaints. Building on this framework, the proposed algorithm seen in Fig. 1 includes neuro-urologic screening questions (Fig. 1a) which if positive may prompt further workup (Fig. 1b). The initial urologic evaluation includes urinalysis, uroflowmetry, post-void residual measurement, and selective bladder diaries, cystoscopy, upper urinary tract imaging, and urodynamic testing.

### Urologist’s Exam

In addition to the standard genitourinary exam seeking e.g. prolapse, prostatomegaly, meatal stenosis, urethral diverticulum, and other anatomic clues a focused neurological exam can be easily noted. Pelvic floor muscle tone and strength can be informative of S2,3,4 segment integrity. Brief assessment of pelvic dermatomal distributions (see Supplemental Fig. 1) by history or exam can be useful in comparing symmetry of sensation (e.g. numbness or pain). The anal sphincter tone and ability to contract should be noted during digital rectal exam (DRE).

Observational assessment during the clinical interaction should take note of apparent focal neurological deficits and other signs of neurological impairment including gait, handshake, speech, or cognitive function. Additional observations could include: cold limbs, venous congestion, and poor capillary refill (may be seen in systemic autonomic dysfunction).

To avoid asking the readers to undertake unfruitful examinations, and given that certain reflexes are not sensitive or specific (e.g. cremasteric) – we are not routinely recommending these as essential to the workup. However, if there is suspicion of a sacral root lesion (e.g. numbness) then the following may be helpful:

Anal reflex: stroke perianal skin with noxious stimulous (e.g. stiff end of Q tip) and observe perianal skin for a contraction (or more reliably feel contraction digitally during DRE).

Bulbocavernosus reflex: more reliable in men. Noxious stimulus to the head of the penis (squeeze) with finger in rectum and observe contraction (evaluates sacral reflex arc).

### Urologist’s Role

Modern urologic practice exists within a healthcare system increasingly constrained by limited subspecialty access. The patient may have waited months for the urology visit and neurology evaluation may involve prolonged wait times of 30–270 days for consultation, delaying the diagnosis of potentially reversible conditions [110]. While awaiting neurological evaluation, the urologist may be comfortable initiating part of the workup for OND. Baseline laboratory testing (see Fig. 1b) can be informative for future neurologic diagnosis or may identify reversible contributors. Urodynamic studies, as detailed below as well as in Fig. 1; Table 1, as well as Supplemental Tables 1 and 2, can help identify features associated with neurologic involvement. Imaging (e.g. MRI) may be ordered by the urologist depending on the index of suspicion and understanding of the appropriate techniques and modalities of their collaborating radiology and neurology colleagues for certain diagnostic questions.

## Urodynamic Findings in Testing Characterization of Occult Neurologic Disease by Symptoms and Urodynamics

OND presents with a wide variety of symptoms based on the extent of the disease and location of the lesion. Urinary symptoms can be categorized into storage or voiding symptoms. *Storage symptoms* occur during the bladder storage phase and include urinary urgency, increased daytime frequency, all types of urinary incontinence, and nocturia [111]. *Voiding symptoms* arise during the bladder emptying phase, and are characterized by urinary hesitancy, intermittency, slow, split, or splayed stream, straining to void, or terminal dribble. Sensation of incomplete bladder emptying and post void dribbling are considered *post-micturition symptoms*. Elevated post void residual is an important sign that can indicate anatomic bladder outlet obstruction, detrusor underactivity, impaired coordination (neurological or functional) between the detrusor and sphincters, or medication or behavioral factors.

### Urodynamic Evaluation

The European Association of Urology (**EAU) Guidelines on Non-Neurogenic Male LUTS** and **Female LUTS**, recommend urodynamics prior to invasive therapy when there is diagnostic uncertainty, poor symptom–anatomy concordance, or concern for non‑obstructive pathology, particularly in men and in patients with atypical presentations. This recommendation is especially relevant at the extremes of age (< 50 and ≥ 80 years), where lower urinary tract symptoms are less likely to reflect fixed outlet obstruction and more likely to represent detrusor dysfunction or occult neurologic disease.

Urodynamic testing is the cornerstone of lower urinary tract evaluation. When interpreted through a neurologic lens, urodynamic findings provide insight into the functional integrity of neural pathways governing micturition, and are particularly useful when symptoms are atypical, refractory, or discordant with anatomic findings [5, 7, 112]. Indications for urodynamic evaluation in the context of suspected neurologic contributors include known neurological disease with established upper urinary tract risk (e.g. spinal cord injury), refractory LUTS despite standard therapy, unexplained storage or voiding dysfunction, urinary retention, suspected bladder outlet obstruction with significant storage component, refractory recurrent urinary tract infections, or clinical suspicion for autonomic nerve involvement [5, 112]. Urodynamics can help distinguish primary urologic disorders (e.g. prostatic obstruction) from underlying neurologic pathology (e.g. non-relaxation of the external urinary sphincter) and guide early diagnostic action. Beyond detection of neurologic patterns, urodynamics also provides practical guidance for clinical management, including tailoring pharmacologic, chemo denervation using botulinum toxin injections, neuromodulation, surgical therapy, and determining upper tract risk and the need for catheterization.

In training, Urologists learn about urodynamic findings with respect to spinal cord level (see Table 1, Supplemental Table 1). However suggestive, none of the following urodynamic findings are pathognomonic for neurological disease.

#### Above the PMC

Losing higher level brain centers above the pontine micturition center (PMC) will lead to varying loss of ability to regulate the PMC. At the more severe end of the spectrum there will be complete loss of control with spontaneous voiding of small volumes, but typically with safe synergistic sphincter parameters due to maintenance of the pathways distal to and including the PMC. In less severe lesions one sees more subtle storage symptoms (frequency) and detrusor overactivity (DO) - the prefrontal cortex and periaqueductal gray are less able to quiet ascending signals and regulate the PMC with respect to social appropriateness.

#### Spinal Cord Above S2

In complete lesions below the PMC and above the S2 sacral spinal cord level (upper lumbar bony level) one would expect loss of PMC regulation via the bulbospinal reticulospinal pathways to the intact sacral reflex arc. Therefore, above S2 one sees disinhibition of the detrusor and lack of relaxation or paradoxical contraction of the external sphincter during voiding (detrusor–external sphincter dyssynergia, DESD). Incontinence and/or retention can both occur depending on this balance, and the upper urinary tracts are at risk due to potential for elevated storage and voiding pressures. Above T6, in addition, one sees bladder neck dyssynergia and autonomic dysreflexia due to sympathetic dysregulation. Reduced bladder compliance can occur due to chronic mechanical stress and intermittent ischemia, due to detrusor overactivity and elevated prolonged voiding pressures, which activate cellular death, repair and fibrosis pathways [113]. Vesicoureteral reflux can occur if the storage and voiding pressure within the bladder overcomes the anti-reflux mechanism at the ureterovesical junction.

#### S2-S4

If a lower spinal cord lesion impacts the S2-S4 spinal cord level, one loses the sacral reflex arc and, hence the bladder contraction, and the external sphincter is underactive due to loss of Onuf’s nucleus. Sacral and infrasacral lesions cause predominantly voiding symptoms due to detrusor underactivity, with urodynamic tracings showing elevated PVR, and an acontractile detrusor [7].

#### Peripheral Pelvic Nerves

If the anatomic peripheral nerves are lost due to pelvic surgery (e.g. the inferior hypogastric plexi during abdominoperineal resection), the sympathetic and parasympathetic input are lost but the external urethral sphincter, which is innervated by the pudendal nerve via Onuf’s nucleus, remains intact. Sacral and infrasacral parasympathetic loss cause predominantly voiding symptoms due to detrusor underactivity, with urodynamic tracings showing elevated PVR, and acontractile detrusor [7].

#### Additional Urodynamic Patterns

Urodynamic patterns are explored in Table 1 below with additional information in Supplemental Tables 1 and 2. Those that may suggest a central nervous system (CNS) lesion include DO, detrusor–external sphincter dyssynergia (DESD), and reduced bladder compliance. Detrusor underactivity and impaired bladder sensation are associated with parasympathetic involvement, as seen in small fiber and other peripheral neuropathies, dysautonomia, surgical disruption of the pelvic nerves, large fiber neuropathies such as MS and injuries impacting the lower spinal cord, as well as myotonic stretch injuries. Bladder neck high-tone dysfunction can indicate bladder neck dyssynergia associated with SCI or other spinal disease above T6, peripheral sympathetic dysfunction, or abnormal autonomic function. An open bladder neck in men during filling in the absence of prior events such as prostate surgery may indicate MSA due to sympathetic nerve dysfunction (Supplemental Table 2d). Detrusor overactivity with sphincter relaxation can be seen in cortical lesions such as stroke or tumor, or in peripheral autonomic nerve dysfunction, or could be simply idiopathic DO. Structural neurologic conditions such as Tarlov cysts, tethered cord, and sacral tumors may produce mixed urodynamic patterns [37, 81].

##### Sphincters

Incomplete emptying or dysuria in the absence of urinary tract infection can result from failure of relaxation at the bladder outlet, involving either the bladder neck (internal urethral sphincter) or the external urethral sphincter.

##### Internal Urethral Sphincter (bladder neck)

Bladder neck or internal sphincter dyssynergia refers to neurogenic failure of bladder neck relaxation during voiding due to abnormal neural control, resulting in functional outlet obstruction in the absence of an anatomic lesion [4]. Spinal lesions above T6, as in MS, spinal cord injury, NMOSD, or transverse myelitis) disrupt descending inhibitory regulation via corticospinal and reticulospinal pathways, permitting unopposed thoracolumbar sympathetic (T11–L2) α-adrenergic activity at the bladder neck via the hypogastric nerve.

Primary bladder neck obstruction has historically been described as a non-neurologic entity. However, peripheral autonomic dysfunction can similarly increase bladder neck tone through abnormal function of unmyelinated C fibers and thinly myelinated Aδ fibers, even in the absence of a central neurologic lesion. In this context, bladder neck obstruction reflects failure of sympathetic withdrawal rather than loss of central micturition coordination. Normally, activation of the pontine micturition center suppresses thoracolumbar α-adrenergic tone, permitting bladder neck relaxation during detrusor contraction. In autonomic neuropathies, impaired inhibitory afferent feedback, denervation hypersensitivity, and dysregulated sympathetic outflow result in persistent or exaggerated bladder neck contraction, producing functional outlet obstruction despite preserved external sphincter relaxation. Notably, the parasympathetic system does not actively “open” the bladder neck; rather, it facilitates voiding by withdrawing sympathetic tone that maintains bladder neck closure. This pattern may be seen in diabetes, small fiber neuropathy, and other peripheral neuropathies with autonomic involvement.

Conversely, a lax bladder neck reflects loss of sympathetic tone and may occur in conditions marked by central autonomic failure, such as multiple system atrophy, which affects thoracolumbar intermediolateral cell columns and sympathetic preganglionic neurons. Similar findings may occur in disorders with peripheral sympathetic denervation, including diabetic autonomic neuropathy, Guillain–Barré syndrome, Charcot–Marie–Tooth disease, and CIDP with autonomic involvement.

##### External Urethral Sphincter

DESD occurs when the bladder (detrusor) contracts while the external urethral sphincter simultaneously and involuntarily contracts instead of relaxing. This happens when the reticulospinal tracts are disrupted, preventing the pontine micturition center from inhibiting Onuf’s nucleus in the sacral spinal cord. This interruption can be caused by spinal cord injury, MS, NMOSD, transverse myelitis, and spinal tumors above the level of the sacral micturition center (S2-4). A lax external sphincter can be caused by damage to Onuf’s nucleus itself (sacral spinal cord injury, MSA, MOGAD) or damage to the peripheral pudendal nerves (diabetes, Guillain-Barre syndrome, advanced CMT, second stage labor, or CIDP). Of note, Amyotrophic Lateral Sclerosis (ALS) usually spares Onuf’s nucleus until late in the disease.

Assessment of findings at the bladder outlet should keep in mind iatrogenic and anatomic etiologies, e.g. history of mid urethral sling, bulking agent, bladder outlet prostate surgery, sympathomimetic medications, obstructing prostate, prolapse, or urethral diverticulum. Functional disorders such as high tone pelvic floor dysfunction mimic neurologic disease, and the testing environment itself may lead to artifactual findings (e.g. the appearance of an atonic bladder when the patient is unable to void on the soft-seated urodynamics chair).

## Multidisciplinary Collaboration

Given the heterogeneity of neurologic contributors to LUTS and systemic constraints on specialty access, close collaboration with neurology is an important mechanism for elevating clinical concern and refining diagnostic pathways. Effective multidisciplinary approaches include electronic medical record-enabled communication, informal consultation, co-management, and structured referral pathways based on symptom clusters [81], or even establishment of a LUTS neuro-urology clinic, similar to existing formats for spina bifida and cerebral palsy. In our experience, establishing working relationships with local neurologists experienced in autonomic and pelvic neurophysiology is an accessible way to address delays in formal neurologic evaluation. Low-threshold, case-based communication allows for early discussion of patients with concerning LUTS, helping to determine whether further neurologic assessment is warranted and which diagnostic studies are appropriate to initiate. Colocalizing in clinic can be a very effective means by which to expedite communication and care. These collaborative interactions facilitate earlier diagnostic alignment, more targeted testing, and timely management. Such relationships are feasible even in settings without formal multidisciplinary infrastructure and support improved diagnostic accuracy and patient outcomes across practice environments. Lastly, urologists often have their own long wait times, short visit duration, and little time for administrative coordination. This algorithm promotes bidirectional cross-specialty collaboration.

## Conclusion

Patients presenting with lower urinary tract symptoms (LUTS) may have occult neurological disease (OND). Whereas Urologists are not expected to make the diagnosis of neurologic disease, it is imperative that they recognize the signs before them, highlighting the possibility and triggering the process for neurological evaluation. We review the urologic presentation of neurological disease with an emphasis on LUTS as a sign of OND. We present a screening tool, an algorithm, and urodynamics guidance for the evaluation of patients presenting with LUTS with respect to when to suspect OND and involve neurology for evaluation. The aim is to enable clinicians to recognize patterns of neurologic disease early.

## Key References

- Drake, M.J., et al., Can We Improve Our Routine Urological Assessment to Exclude Neurogenic Causes for Lower Urinary Tract Dysfunction? ICI-RS 2024. Neurourol Urodyn, 2025. 44(3): p. 609–615.
  - ○This reference highlights the importance and challenges of identifying occult neurologic disease in patients presenting with LUTS, emphasizing the need for multidisciplinary evaluation, thorough neuro-urologic physical exam, and integration of urodynamic studies to allow for early identification.
- Roy, H.A., et al., Assessment of patients with lower urinary tract symptoms where an undiagnosed neurological disease is suspected: A report from an International Continence Society consensus working group. Neurourol Urodyn, 2020. 39(8): p. 2535–2543.
  - ○This reference includes the most common neurological conditions which present with LUTS early in the disease course and recommendations for prompt clinical identification.
- Vichayanrat, E., et al., Lower urinary tract dysfunction reported in autonomic disorders. Auton Neurosci, 2025. 262: p. 103341.
  - ○This reference outlines the clinical features and common presentations of autonomic neurologic disorders, along with a list of key autonomic conditions relevant to urologic practice.
- Panicker, J.N., et al., European Academy of Neurology (EAN)/European Federation of Autonomic Societies (EFAS)/International Neuro-Urology Society (INUS) Guidelines for Practising Neurologists on the Assessment and Treatment of Neurogenic Urinary and Sexual Symptoms (NEUROGED Guidelines). Eur J Neurol, 2025. 32(4): p. e70119.
  - ○This reference provides updated guidelines, including 38 NEUROGED recommendations, to guide neurologists in the comprehensive evaluation of urinary and sexual symptoms in patients with neurologic diseases.”

## Supplementary Information

Below is the link to the electronic supplementary material.

Supplemental File 1 (PPTX 4.80 MB)

## Data Availability

No datasets were generated or analysed during the current study.

## Declarations

MD is on speaker bureau and advisory boards for Astellas, Pierre Fabre, Nowwell, and Viatris.

ED is Chair DSMB NIDDK/NIH Protocol Number: EPPIC22001, a consultant for Flume catheters and Luca Biologics, is on the advisory board for Ironwood Pharmaceuticals and Glycologix, was PI on a study for Ironwood pharmaceuticals, has stock in Doximity and ERYP, Shares in Restoration Medicine, Owner of Pelvic Answers LLC and runs www.facingpelvicpain.org.
