# Supplementary material for: Lower Urinary Tract Symptoms as an Indicator of Occult Neurologic Disease: A System-first Framework for Urologic Practice
Source: Curr Urol Rep. 2026 Jun 2;27(1):28. doi: 10.1007/s11934-026-01343-2 (PMC13230311; doi:10.1007/s11934-026-01343-2)
Supplement: Supplementary file 1 — Supplemental File 1 (PPTX 4.80 MB) [file 11934_2026_1343_MOESM1_ESM.pptx]

## Slide 1
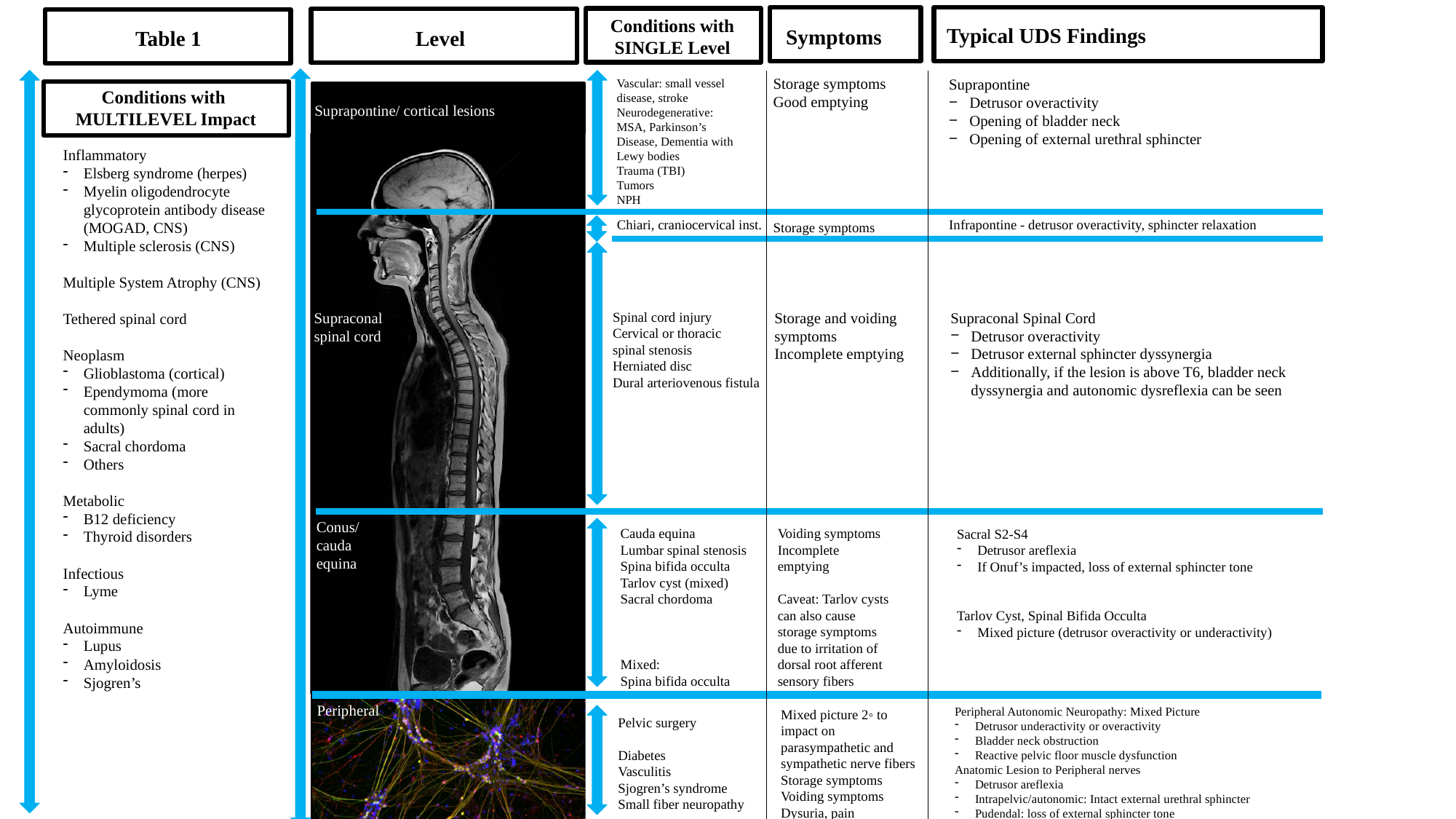

Conditions with SINGLE Level
Typical UDS Findings
Symptoms
Level
Table 1
Storage symptoms
Good emptying
Suprapontine
Detrusor overactivity
Opening of bladder neck
Opening of external urethral sphincter
Vascular: small vessel disease, stroke
Neurodegenerative: MSA, Parkinson’s Disease, Dementia with Lewy bodies
Trauma (TBI)
Tumors
NPH
Conditions with
MULTILEVEL Impact
Suprapontine/ cortical lesions
Inflammatory
Elsberg syndrome (herpes)
Myelin oligodendrocyte glycoprotein antibody disease (MOGAD, CNS)
Multiple sclerosis (CNS)
Multiple System Atrophy (CNS)
Tethered spinal cord
Neoplasm
Glioblastoma (cortical)
Ependymoma (more commonly spinal cord in adults)
Sacral chordoma
Others
Metabolic
B12 deficiency
Thyroid disorders
Infectious
Lyme
Autoimmune
Lupus
Amyloidosis
Sjogren’s
Infrapontine - detrusor overactivity, sphincter relaxation
Chiari, craniocervical inst.
Storage symptoms
Storage and voiding symptoms
Incomplete emptying
Supraconal Spinal Cord
Detrusor overactivity
Detrusor external sphincter dyssynergia
Additionally, if the lesion is above T6, bladder neck dyssynergia and autonomic dysreflexia can be seen
Spinal cord injury
Cervical or thoracic
spinal stenosis
Herniated disc
Dural arteriovenous fistula
Supraconal
spinal cord
Conus/
cauda
equina
Voiding symptoms
Incomplete emptying
Caveat: Tarlov cysts can also cause storage symptoms due to irritation of dorsal root afferent sensory fibers
Cauda equina
Lumbar spinal stenosis
Spina bifida occulta
Tarlov cyst (mixed)
Sacral chordoma
Mixed:
Spina bifida occulta
Sacral S2-S4
Detrusor areflexia
If Onuf’s impacted, loss of external sphincter tone
Tarlov Cyst, Spinal Bifida Occulta
Mixed picture (detrusor overactivity or underactivity)
Peripheral
Peripheral Autonomic Neuropathy: Mixed Picture
Detrusor underactivity or overactivity
Bladder neck obstruction
Reactive pelvic floor muscle dysfunction
Anatomic Lesion to Peripheral nerves
Detrusor areflexia
Intrapelvic/autonomic: Intact external urethral sphincter
Pudendal: loss of external sphincter tone
Mixed picture 2◦ to impact on parasympathetic and sympathetic nerve fibers
Storage symptoms
Voiding symptoms
Dysuria, pain
Pelvic surgery
Diabetes
Vasculitis
Sjogren’s syndrome
Small fiber neuropathy

## Slide 2
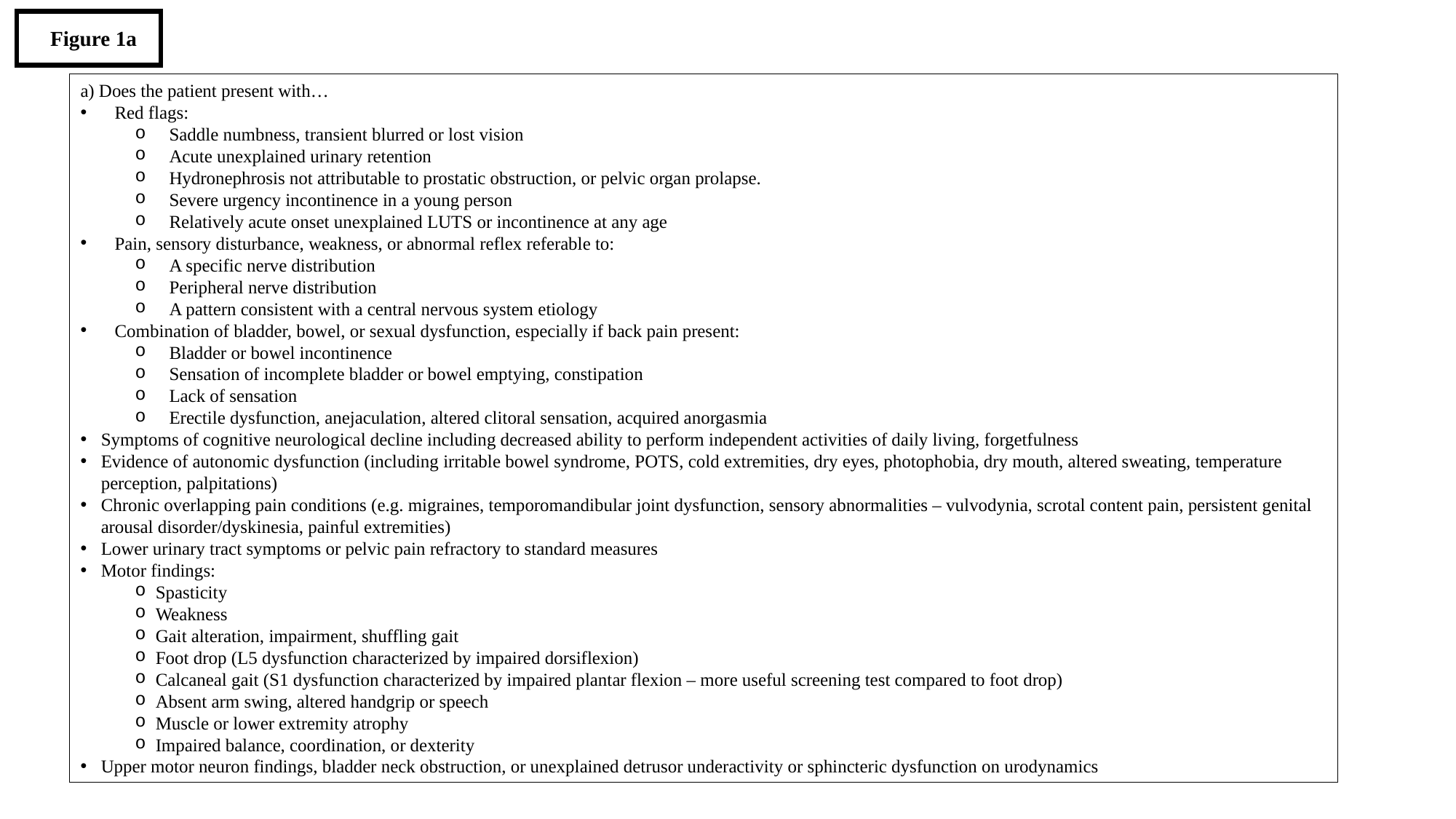

Figure 1a
a) Does the patient present with…
Red flags:
Saddle numbness, transient blurred or lost vision
Acute unexplained urinary retention
Hydronephrosis not attributable to prostatic obstruction, or pelvic organ prolapse.
Severe urgency incontinence in a young person
Relatively acute onset unexplained LUTS or incontinence at any age
Pain, sensory disturbance, weakness, or abnormal reflex referable to:
A specific nerve distribution
Peripheral nerve distribution
A pattern consistent with a central nervous system etiology
Combination of bladder, bowel, or sexual dysfunction, especially if back pain present:
Bladder or bowel incontinence
Sensation of incomplete bladder or bowel emptying, constipation
Lack of sensation
Erectile dysfunction, anejaculation, altered clitoral sensation, acquired anorgasmia
Symptoms of cognitive neurological decline including decreased ability to perform independent activities of daily living, forgetfulness
Evidence of autonomic dysfunction (including irritable bowel syndrome, POTS, cold extremities, dry eyes, photophobia, dry mouth, altered sweating, temperature perception, palpitations)
Chronic overlapping pain conditions (e.g. migraines, temporomandibular joint dysfunction, sensory abnormalities – vulvodynia, scrotal content pain, persistent genital arousal disorder/dyskinesia, painful extremities)
Lower urinary tract symptoms or pelvic pain refractory to standard measures
Motor findings:
Spasticity
Weakness
Gait alteration, impairment, shuffling gait
Foot drop (L5 dysfunction characterized by impaired dorsiflexion)
Calcaneal gait (S1 dysfunction characterized by impaired plantar flexion – more useful screening test compared to foot drop)
Absent arm swing, altered handgrip or speech
Muscle or lower extremity atrophy
Impaired balance, coordination, or dexterity
Upper motor neuron findings, bladder neck obstruction, or unexplained detrusor underactivity or sphincteric dysfunction on urodynamics

## Slide 3
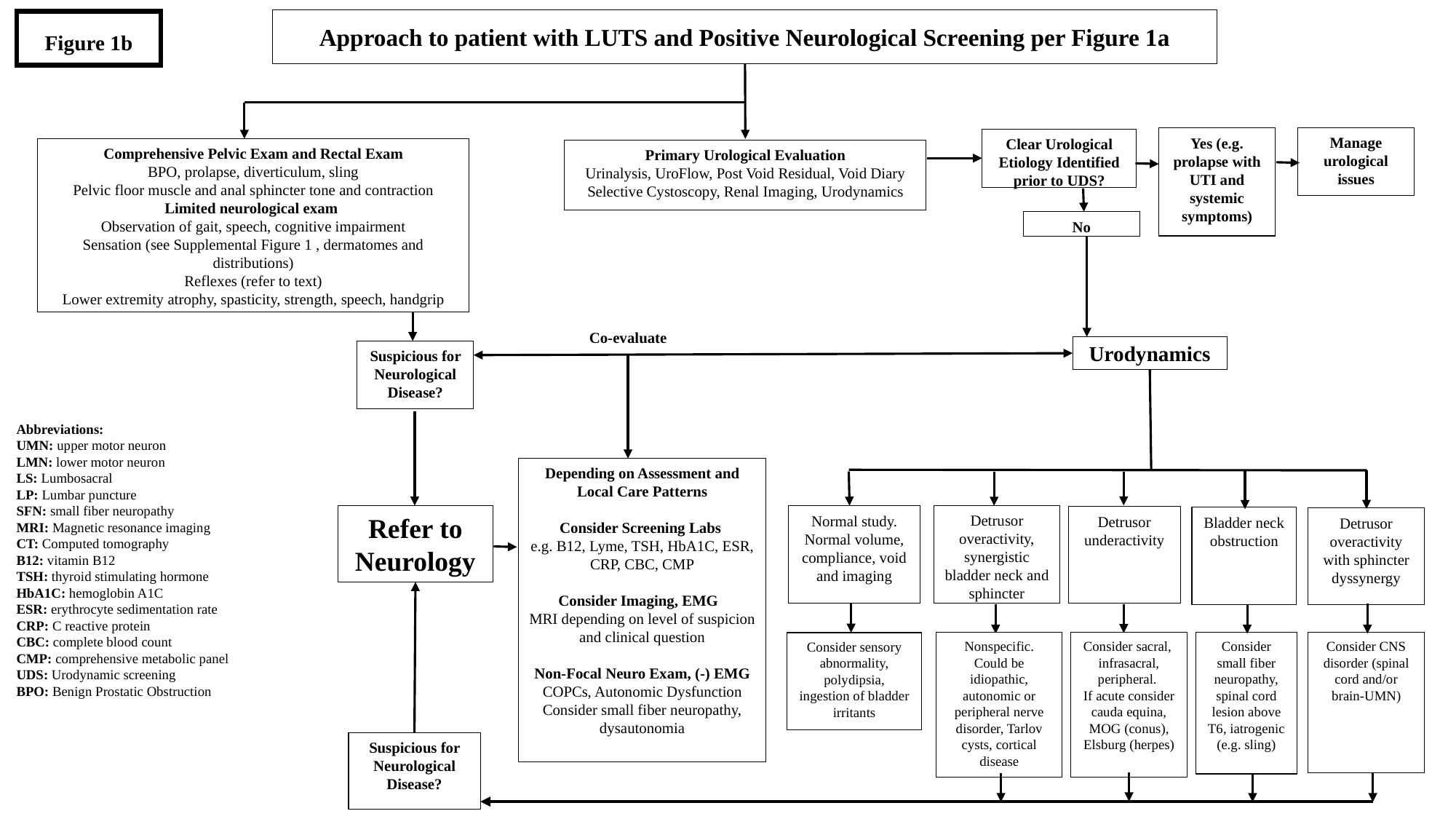

Approach to patient with LUTS and Positive Neurological Screening per Figure 1a
Comprehensive Pelvic Exam and Rectal Exam
BPO, prolapse, diverticulum, sling
Pelvic floor muscle and anal sphincter tone and contraction
Limited neurological exam
Observation of gait, speech, cognitive impairment
Sensation (see Supplemental Figure 1 , dermatomes and distributions)
Reflexes (refer to text)
Lower extremity atrophy, spasticity, strength, speech, handgrip
Primary Urological Evaluation
Urinalysis, UroFlow, Post Void Residual, Void Diary
Selective Cystoscopy, Renal Imaging, Urodynamics
Urodynamics
Abbreviations:
UMN: upper motor neuron
LMN: lower motor neuron
LS: Lumbosacral
LP: Lumbar puncture
SFN: small fiber neuropathy
MRI: Magnetic resonance imaging
CT: Computed tomography
B12: vitamin B12
TSH: thyroid stimulating hormone
HbA1C: hemoglobin A1C
ESR: erythrocyte sedimentation rate
CRP: C reactive protein
CBC: complete blood count
CMP: comprehensive metabolic panel
UDS: Urodynamic screening
BPO: Benign Prostatic Obstruction
Depending on Assessment and Local Care Patterns
Consider Screening Labs​
e.g. B12, Lyme, TSH, HbA1C, ESR, CRP, CBC, CMP
Consider Imaging, EMG
MRI depending on level of suspicion and clinical question
Non-Focal Neuro Exam, (-) EMG COPCs, Autonomic Dysfunction
Consider small fiber neuropathy, dysautonomia
Refer to Neurology
Normal study. Normal volume, compliance, void and imaging
Detrusor underactivity
Detrusor overactivity with sphincter dyssynergy
Consider CNS disorder (spinal cord and/or brain-UMN)
Consider sensory abnormality, polydipsia, ingestion of bladder irritants
Figure 1b
Manage urological issues
Yes (e.g. prolapse with UTI and systemic symptoms)
Clear Urological Etiology Identified prior to UDS?
No
Co-evaluate
Suspicious for Neurological Disease?
Detrusor overactivity, synergistic bladder neck and sphincter
Bladder neck obstruction
Consider sacral,
infrasacral, peripheral.
If acute consider cauda equina, MOG (conus), Elsburg (herpes)
Consider small fiber neuropathy, spinal cord lesion above T6, iatrogenic (e.g. sling)
Nonspecific. Could be idiopathic, autonomic or peripheral nerve disorder, Tarlov cysts, cortical disease
Suspicious for Neurological Disease?

## Slide 4
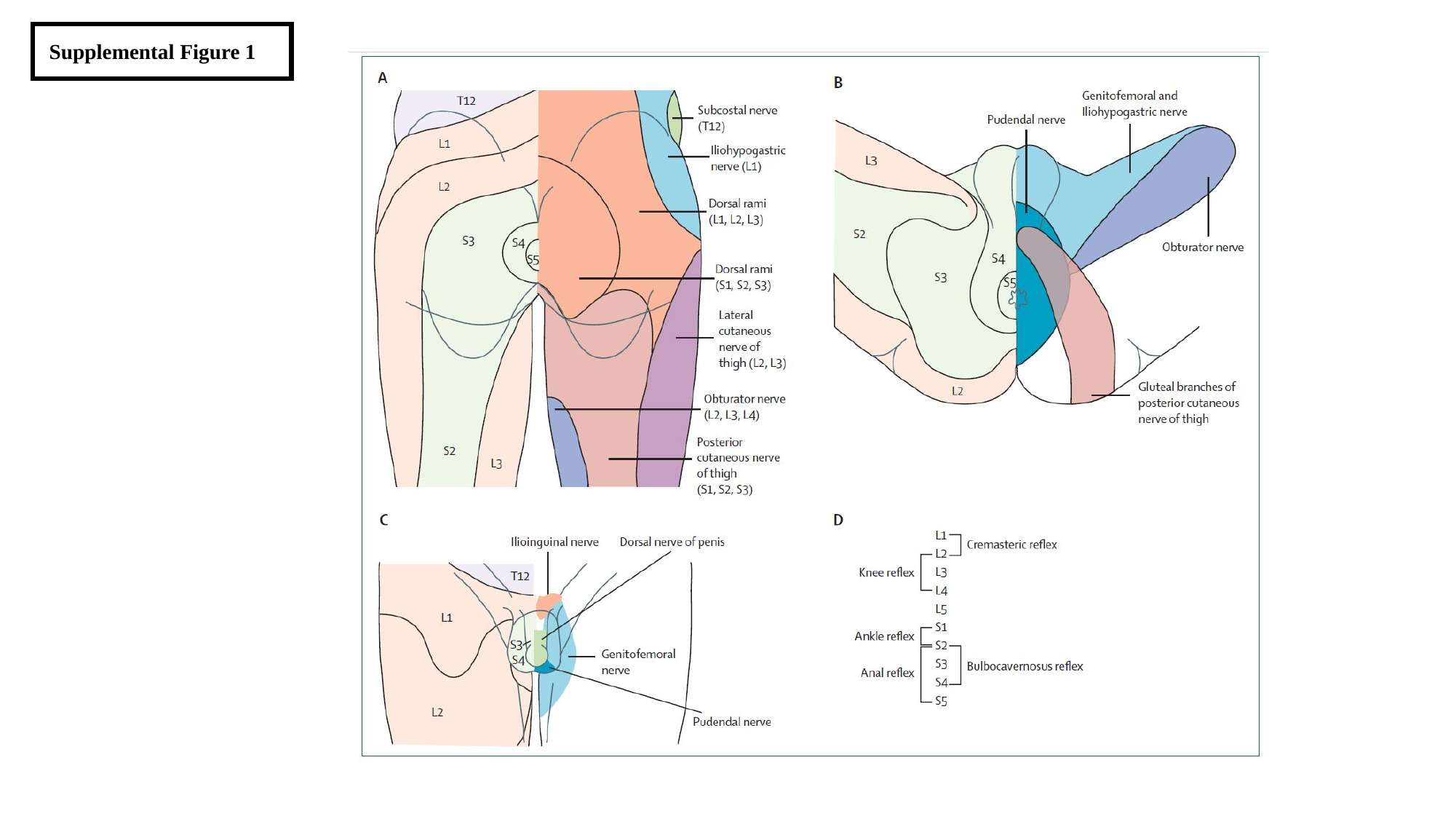

Supplemental Figure 1

## Slide 5
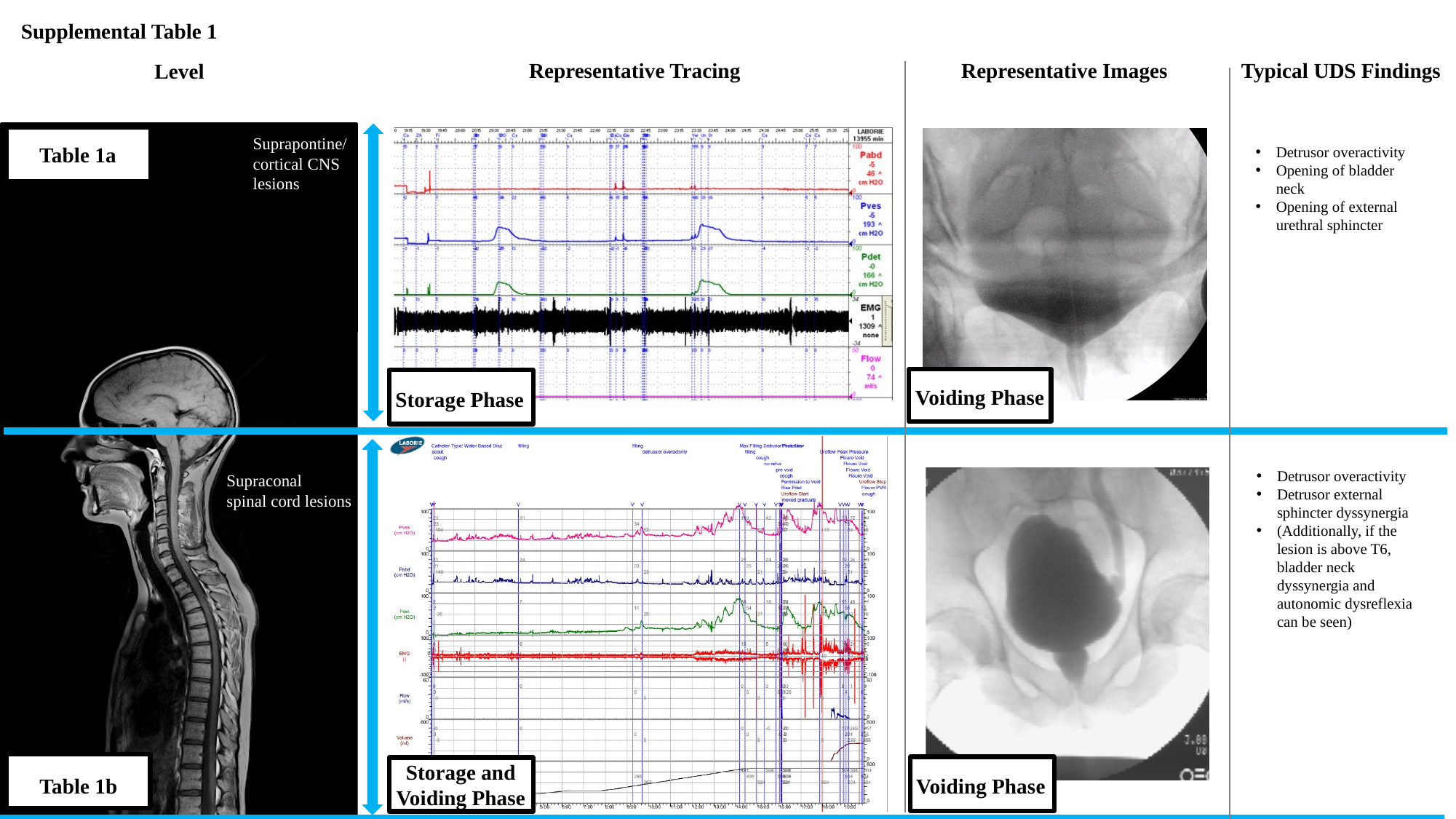

Supplemental Table 1
Typical UDS Findings
Representative Tracing
Representative Images
Level
Suprapontine/ cortical CNS lesions
Table 1a
Detrusor overactivity
Opening of bladder neck
Opening of external urethral sphincter
Voiding Phase
Storage Phase
Detrusor overactivity
Detrusor external sphincter dyssynergia
(Additionally, if the lesion is above T6, bladder neck dyssynergia and autonomic dysreflexia can be seen)
Supraconal
spinal cord lesions
Storage and Voiding Phase
Voiding Phase
Table 1b

## Slide 6
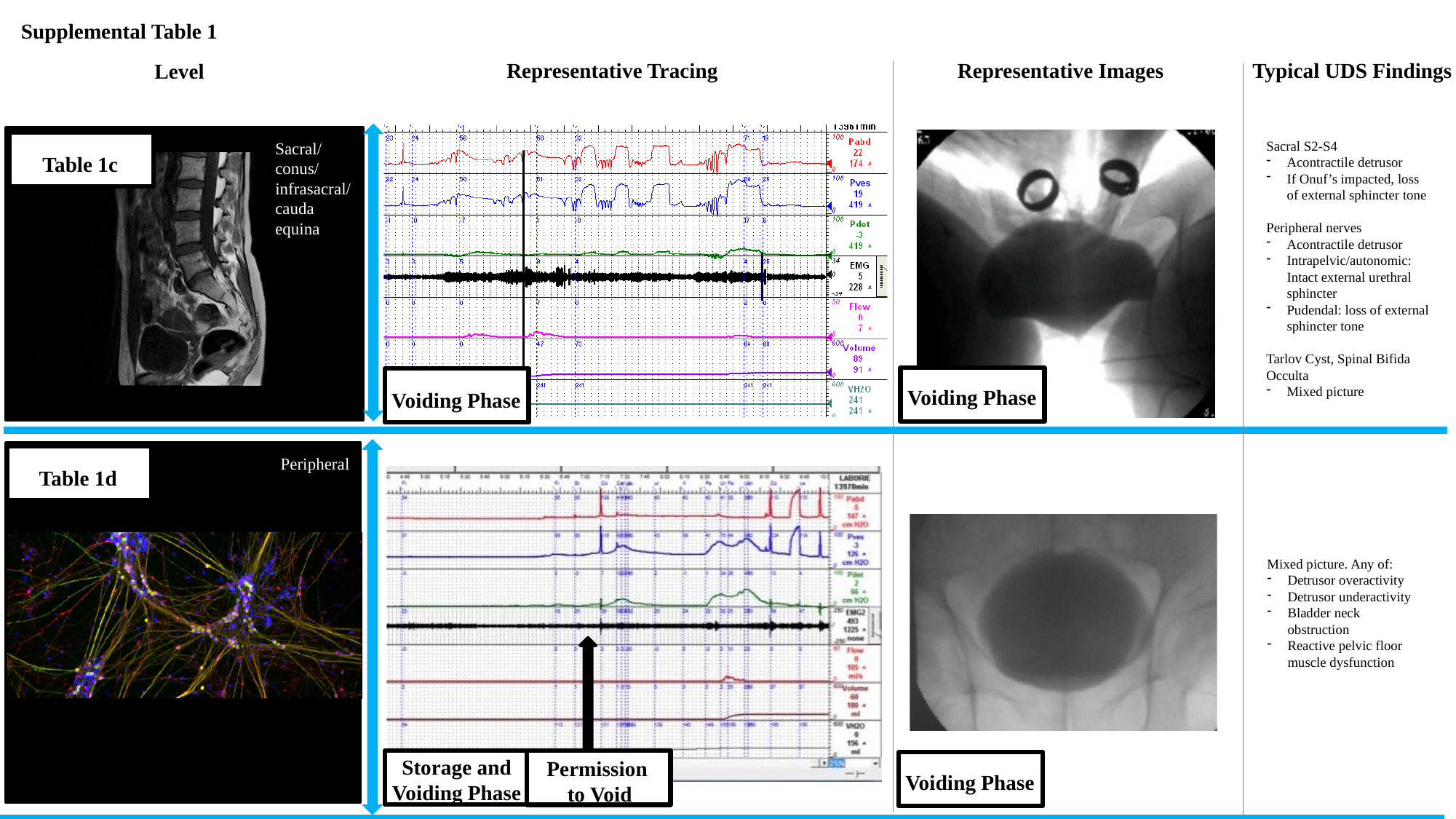

Supplemental Table 1
Typical UDS Findings
Representative Tracing
Representative Images
Level
Sacral S2-S4
Acontractile detrusor
If Onuf’s impacted, loss of external sphincter tone
Peripheral nerves
Acontractile detrusor
Intrapelvic/autonomic: Intact external urethral sphincter
Pudendal: loss of external sphincter tone
Tarlov Cyst, Spinal Bifida Occulta
Mixed picture
Sacral/
conus/
infrasacral/
cauda
equina
Table 1c
Voiding Phase
Voiding Phase
Peripheral
Table 1d
Peripheral
Mixed picture. Any of:
Detrusor overactivity
Detrusor underactivity
Bladder neck obstruction
Reactive pelvic floor muscle dysfunction
Storage and Voiding Phase
Permission
to Void
Voiding Phase

## Slide 7
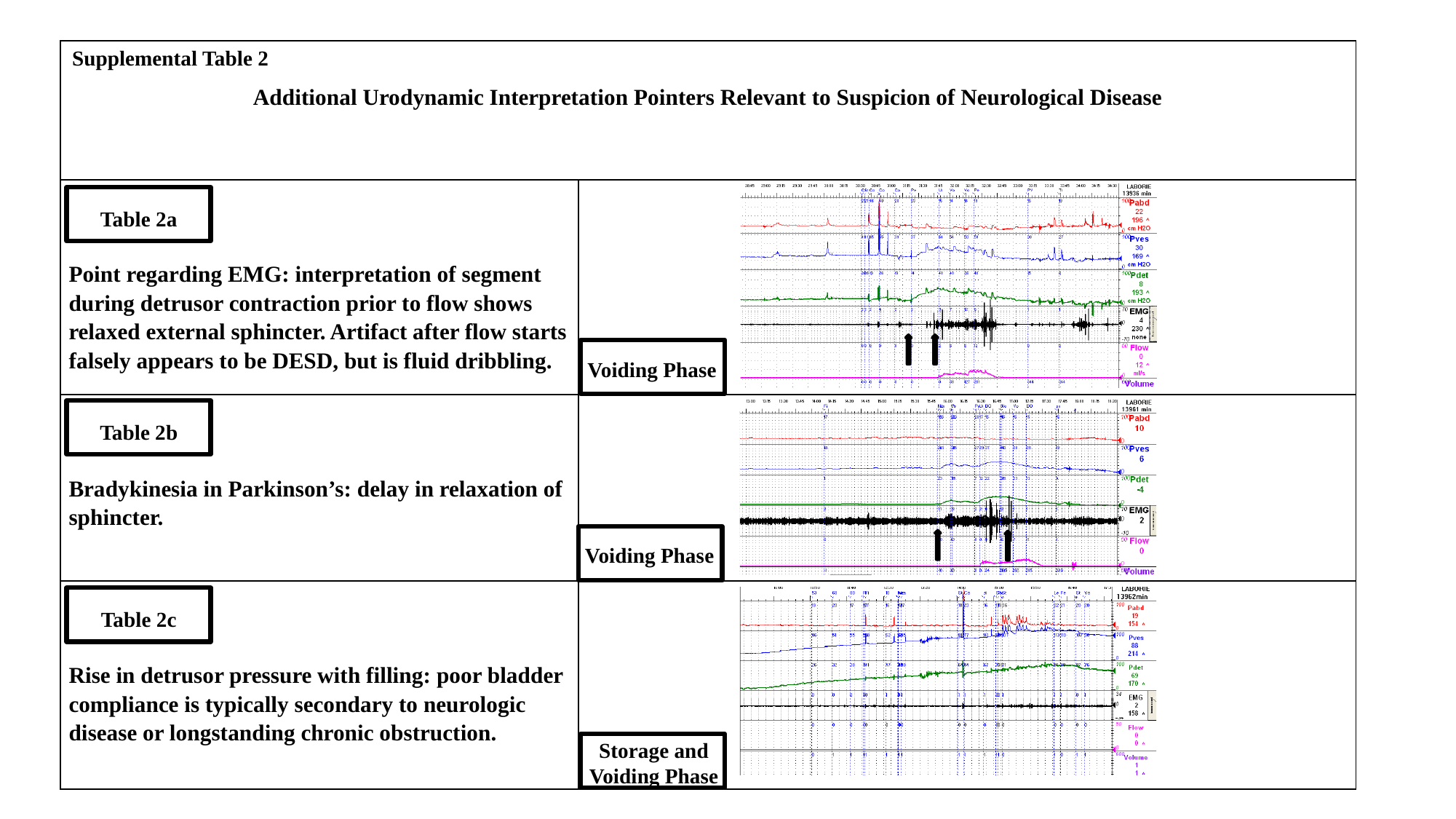

Supplemental Table 2
| Additional Urodynamic Interpretation Pointers Relevant to Suspicion of Neurological Disease | |
| --- | --- |
| Point regarding EMG: interpretation of segment during detrusor contraction prior to flow shows relaxed external sphincter. Artifact after flow starts falsely appears to be DESD, but is fluid dribbling. | |
| Bradykinesia in Parkinson’s: delay in relaxation of sphincter. | |
| Rise in detrusor pressure with filling: poor bladder compliance is typically secondary to neurologic disease or longstanding chronic obstruction. | |
Table 2a
Voiding Phase
Table 2b
Voiding Phase
Table 2c
Storage and Voiding Phase

## Slide 8
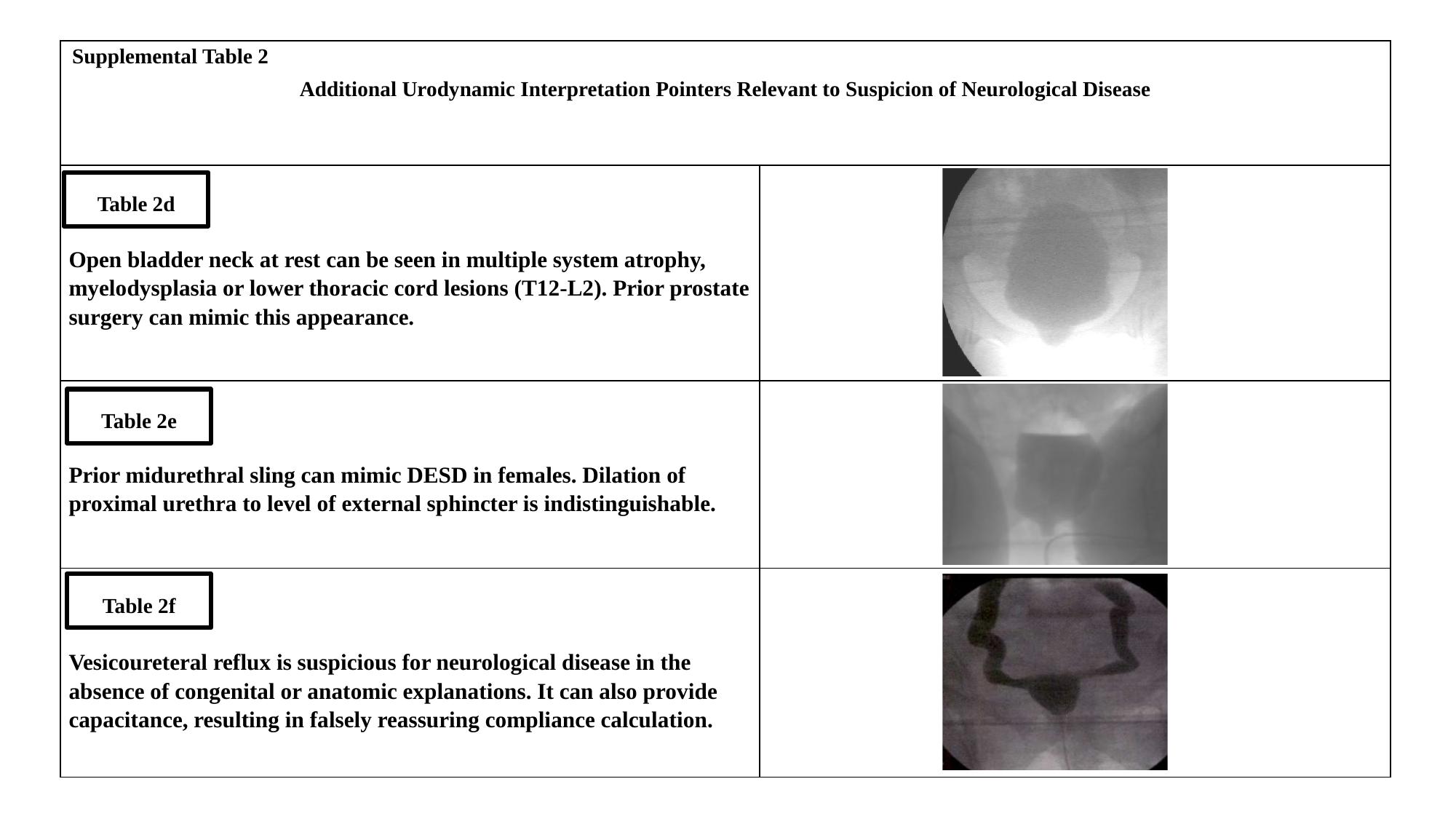

Supplemental Table 2
| Additional Urodynamic Interpretation Pointers Relevant to Suspicion of Neurological Disease | |
| --- | --- |
| Open bladder neck at rest can be seen in multiple system atrophy, myelodysplasia or lower thoracic cord lesions (T12-L2). Prior prostate surgery can mimic this appearance. | |
| Prior midurethral sling can mimic DESD in females. Dilation of proximal urethra to level of external sphincter is indistinguishable. | |
| Vesicoureteral reflux is suspicious for neurological disease in the absence of congenital or anatomic explanations. It can also provide capacitance, resulting in falsely reassuring compliance calculation. | |
Table 2d
Table 2e
Table 2f
